# PhoU homologs from *Staphylococcus aureus* dimerization and protein interactions

**DOI:** 10.1128/spectrum.02067-24

**Published:** 2024-12-11

**Authors:** Clayton T. Matthews, Sakib Mahmud, Stewart G. Gardner

**Affiliations:** 1 School of Science + Mathematics, Emporia State University, Emporia, Kansas, USA; McGill University, Ste-Anne-de-Bellevue, Quebec, Canada

**Keywords:** PhoU1, PhoU2, PitR, *Staphylococcus aureus*, BACTH, dimerization

## Abstract

PhoU proteins are negative regulators of the phosphate response, regulate virulence, and contribute to antibiotic resistance. *Staphylococcus aureus* has multiple genes encoding PhoU homologs that regulate persister formation and potentially virulence, but the molecular mechanisms of this regulation are not fully understood. We used a bacterial adenylate cyclase two-hybrid system to assess interactions between PhoU homologs and other proteins known to interact with PhoU from *Escherichia coli. S. aureus* PhoU (also referred to as PhoU1) interacted with PhoU itself; PitR (also referred to as PhoU2) interacted with PitR itself. We identified potential structural and dimerization models for *S. aureus* PhoU homologs. Dimerization was confirmed using size exclusion chromatography of purified proteins. These results highlight the complex nature of PhoU proteins. Further analysis may elucidate the potential mechanisms for regulating gene expression, persister formation, and virulence in *S. aureus*.

**IMPORTANCE:**

PhoU proteins affect pathogenesis and persister formation in many bacterial species. This protein is essential for signaling environmental phosphate levels in *Escherichia coli* but is still not well characterized in many other pathogenic bacterial strains. This work identifies some similarities and key differences in *Staphylococcus aureus* PhoU homologs compared to *E. coli* PhoU, specifically, PhoU and PitR from *S. aureus* form homodimers but do not appear to interact with PhoR or phosphate transporter proteins.

## INTRODUCTION

PhoU proteins from various bacterial species are important negative regulators of the phosphate response, adapting to changes in environmental phosphate availability (1–4). Regulation occurs through modulating phosphate transport and signaling through a phosphate response two-component signaling system (2, 5–7). In *Escherichia coli*, PhoR histidine kinase controls the phosphate response through adding or removing phosphate from the response regulator, PhoB. However, PhoR lacks any significant extracytoplasmic domains to directly sense environmental phosphate levels (8). Instead, PhoR receives a signal through interaction with PhoU which also interacts with the PstB portion of the high-affinity phosphate-specific transporter complex, composed of the PstS, PstC, PstA, and PstB proteins (PstSCAB) (5–7).

The mechanisms for regulating the phosphate response are not universal across all species of bacteria. For example, in the *Bacillus subtilis* (with no identified PhoU-encoding genes), wall teichoic acid intermediates containing phosphate are sensed through the cytoplasmic Per-Arnt-Sim domain of PhoR as an indicator of phosphate availability, and cell wall subunit synthesis/degradation is regulated as a way to adapt to changes in available phosphate levels (9, 10). Other results in *Salmonella enterica* point to intracellular phosphate levels being the signal that controls PhoR/PhoB activation (11).

Phosphate response proteins also appear to regulate virulence factors and affect antibiotic susceptibility in various bacterial species. For instance, PhoY (a PhoU homolog) in *Mycobacterium tuberculosis* promotes the formation of persisters, a small portion of cells in a population with less sensitivity to an antibiotic (12). PhoU is important for biofilm development in *Mycobacterium smegmatis* (1). The PstSCAB transporter proteins are essential for capsule production in *Streptococcus pneumoniae* (3). PhoU2 is an important regulator in biofilm formation and stress tolerance *in Staphylococcus epidermidis* (13). Another recent study in *Salmonella enterica* identified regulatory roles of PhoU, both in repressing the phosphate response in high phosphate conditions and inducing PhoR activation in low Mg^2+^ conditions (14). Additionally, a *S. enterica* strain with a mutated *phoB*/*phoR* operon was used as a vaccine candidate in chickens, highlighting the potential for these systems to be used as a target for the control of microbial growth and future disease treatment and prevention (15). Recently, the PhoBR system of *Aeromonas dhakensis* was identified as a critical regulator of virulence in zebrafish (16). *Staphylococcus aureus* is a dangerous human pathogen, and while PhoU proteins in *S. aureus* have been studied, the molecular mechanisms of PhoU regulation in *S. aureus* remain unclear (17, 18). There are three genes identified in *S. aureus* that encode *phoU*-like sequences (19). These include genes encoding two PhoU homologs: *phoU* (also known as *phoU1*), located next to the *pstSCAB* genes (similar to *phoU* in *E. coli*), and *pitR* (also known as *phoU2*), found next to the inorganic phosphate transporter gene *pitA*. Additionally, the *nptA* gene encodes for a sodium and phosphate transporter that contains a PhoU-like domain (19).

PhoU is not the dominant negative regulator of the phosphate response in *S. aureus* (20). In *E. coli*, PhoU loss-of-function mutants show a severe growth defect in the presence of excess phosphate (21). However, deleting *phoU*, *pitR*, and both *phoU* and *pitR* genes in the same strain of *S. aureus* did not cause a growth defect but led to the loss of some antibiotic resistance and lower stress tolerance (17). NptA in *S. aureus* improves phosphate uptake and enhances pathogenesis (19). All three phosphate transporters (PstSCAB, PitA, and NptA) have overlapping but not redundant functions in *S. aureus* (19). PhoU can limit excess transport of phosphate by the PstSCAB complex for cells growing in high-phosphate conditions for some bacteria under certain conditions (2).

Interestingly, one study found that a point mutation in the *pitA* gene led to nonsusceptibility to the antibiotic daptomycin and elevated concentrations of intracellular phosphate, but this phenotype was lost when the upstream gene *pitR* was inactivated (22). This complex relationship underscores the multifaceted functions of the PhoU homologs in *S. aureus*. Crystal structure studies identified a similar structure for various PhoU proteins. Some studies identified PhoU protein monomers, while other studies found dimer formation of two PhoU proteins (23–25). Still, other structures show trimer and even hexamer formation of PhoU proteins (26, 27). In *E. coli*, PhoU/PhoU interaction was determined using a bacterial two-hybrid system, and purified PhoU proteins eluted at the expected size of a dimer from a size exclusion column (6). Clarifying the structures of *S. aureus* PhoU proteins may shed light on their molecular function in signaling.

This study seeks to better understand the molecular mechanisms of PhoU homologs in *S. aureus* by identifying protein/protein interactions using a bacterial adenylate cyclase two-hybrid system (BACTH), modeling protein structures, predicting dimerization interactions, and confirming dimerization with size-exclusion chromatography. These results provide evidence of similarities and differences between *S. aureus* PhoU proteins and PhoU proteins from other bacteria.

## RESULTS

### BACTH interactions

**Fig 1:**
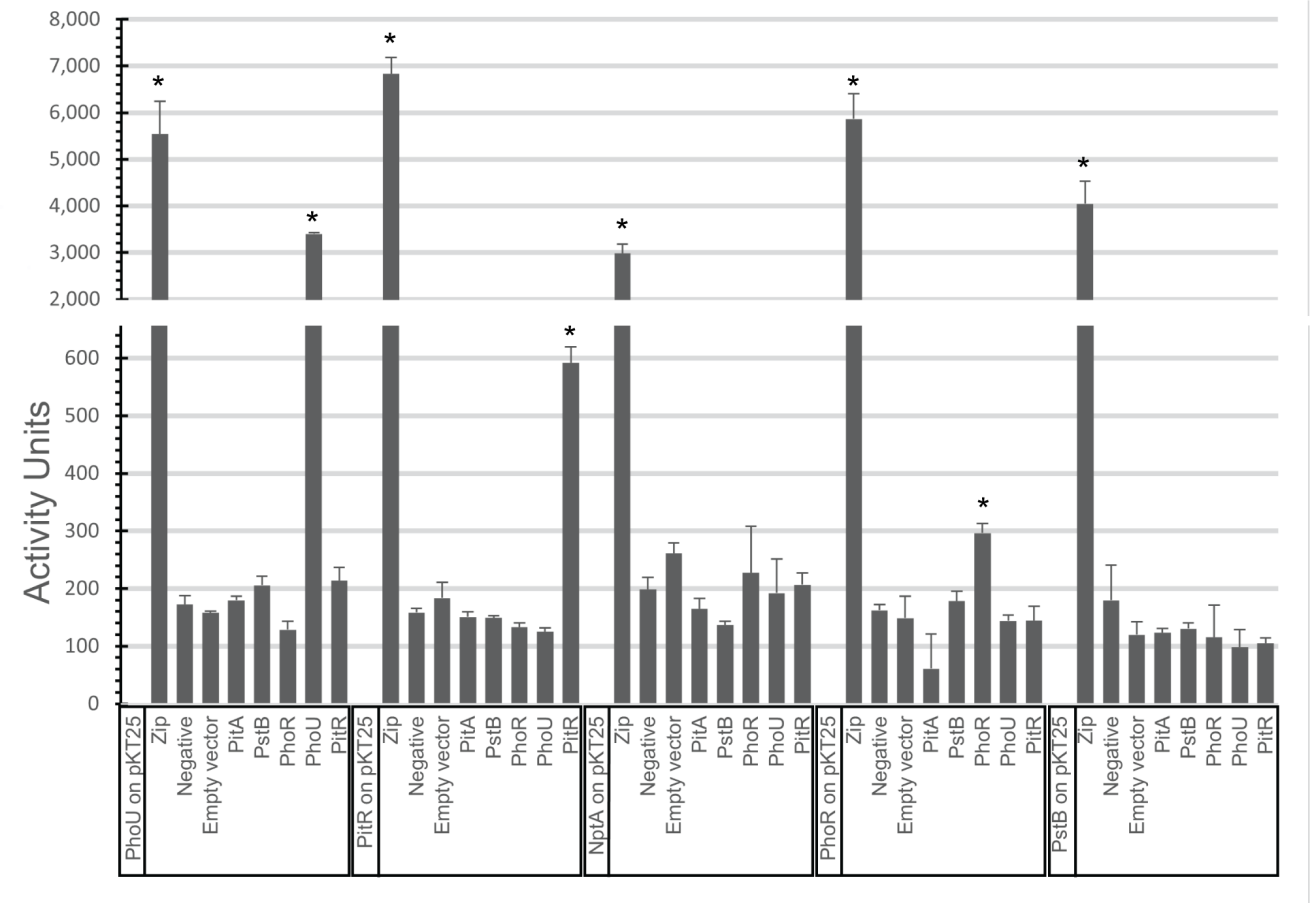
BACTH results. β-galactosidase activity for each combination of proteins in the BACTH system. Samples are grouped with the different proteins expressed from the pKT25 plasmid in combination with other proteins expressed from the pUT18C plasmid. The samples labeled Zip represent the positive control of leucine zipper domain genes cloned into both plasmids, the Negative label represents no gene cloned into the pUT18C plasmid, and samples labeled Empty have no genes cloned into either the pKT25 or pUT18C plasmids. Each sample shows the mean ± SD of four biological replicates (with three technical replications for each biological replicate). Samples were analyzed with a Dunnett’s multiple comparisons test compared to the negative control sample. Samples with significantly more activity are identified, *P*-values < 0.001 with *.

When expressed from the pKT25 plasmid, PhoU showed significant interaction with PhoU expressed from the pUT18C plasmid. There was no significant interaction seen between PhoU and PhoR (Fig. 1). When PitR from pKT25 was tested, it showed significant interaction with PitR. PitR did not significantly interact with PhoU, PstB, or PhoR. Tests of NptA did not show any significant interactions with the other PhoU homologs, PstB, or PhoR proteins. The other phosphate transporter in *S. aureus*, PitA, did not show any significant interaction with any of the other proteins tested (Fig. 1).

Tests were also conducted using PstB and PhoR expressed from the pKT25 plasmid. The only significant interaction observed, other than the positive controls, was that PhoR interacted with PhoR, which was expected given the dimer nature of PhoR (Fig. 1).

### Protein structure modeling

**Fig 2:**
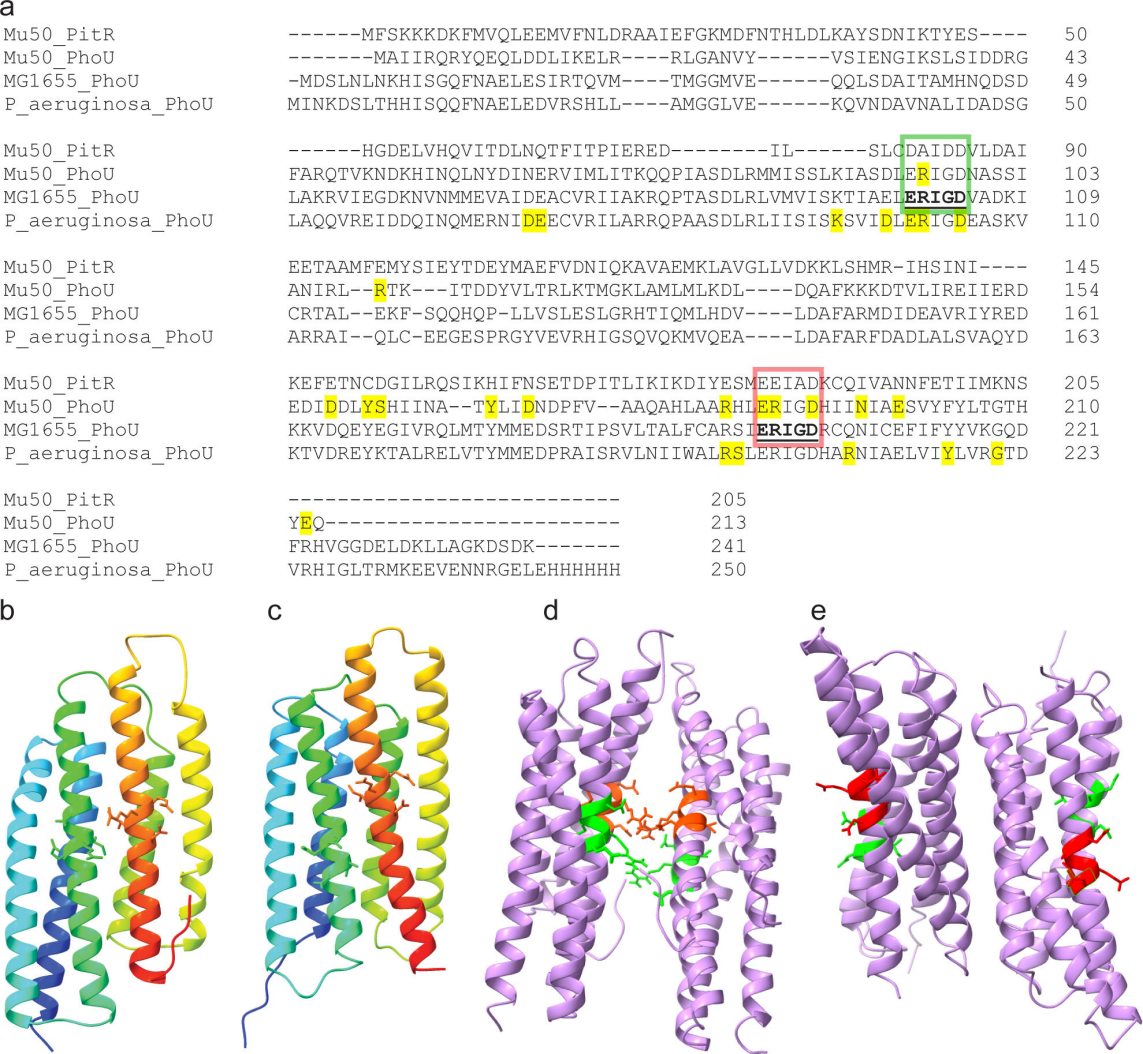
PhoU- and PitR-predicted structures. (**a**) Alignments of PhoU amino acid sequences from *S. aureus* (Mu50_PhoU and Mu50_PitR), *E. coli* (MG1655), and *P. aeruginosa* (P_aeruginosa_PhoU). The PhoU conserved residues of the *E. coli* sequence are in bold and underlined. Amino acids highlighted in the structure images (Fig. 2b through e) are shown with green and red boxes. Residues involved in hydrogen bonding between PhoU proteins are highlighted yellow based on the crystal structure of *P. aeruginosa* PhoU (23) and the dimer model for *S. aureus* PhoU. (**b**) *Staphylococcus aureus* PhoU-predicted protein structure. (**c**) PitR-predicted protein structure. Images are colored red to blue from N to C terminus. (**d**) PhoU homodimer model. (**e**) PitR homodimer model.

The protein sequences of *S. aureus* PhoU and PitR were aligned with the sequence of PhoU from *E. coli* and PhoU from *P. aeruginosa* to identify conserved residues in the sequences (Fig. 2a). PhoU and PitR protein sequences from *S. aureus* were analyzed using Protein Homology/analogY Recognition Engine V 2.0 (28). Both proteins were predicted to have the classic PhoU structure of two bundles of three alpha helices, and conserved amino acid residues are found on the same face of the predicted structures (Fig. 2b and c). The PhoU structure model was based on the template d1sumb (the PhoU protein from *Thermotoga maritima* http://scop.berkeley.edu/pdb/code=1sum) with 100.0% confidence and 97% coverage, modeling 207 residues for *S. aureus* PhoU. The PitR structure model was based on the template c3l39A (the PhoU protein from *Bacteroides thetaiotaomicron* https://www.rcsb.org/structure/3l39) with 100.0% confidence and 100% coverage, modeling 204 residues for *S. aureus* PitR.

To identify potential *S. aureus* PhoU dimerization structure models, the predicted PhoU structures were used to predict interaction models with the Cluspro 2.0 protein-protein docking tool (29). Several models were generated for each interaction. We show models that appear to match other known PhoU dimer structures (Fig. 2d and e).

The PhoU/PhoU dimer model has the monomers aligned with the two conserved ERXXD regions of the proteins facing each other (Fig. 2d). The PitR/PitR dimer model has the proteins rotated so the protein faces with the conserved regions oriented away from the dimer interface (Fig. 2e).

In the predicted dimerization models, several hydrogen bonds are found between the PhoU/PhoU dimer but not in the PitR/PitR dimer model. The residues involved in hydrogen bond formation found in the crystal structure for *P. aeruginosa* PhoU partially correlate with residues involved in the predicted hydrogen bond interactions of the *S. aureus* PhoU homodimer model (23). In the PitR sequence, several residues involved in hydrogen bond formation do not appear to be conserved (Fig. 2a).

### Protein expression and characterization

**Fig 3:**
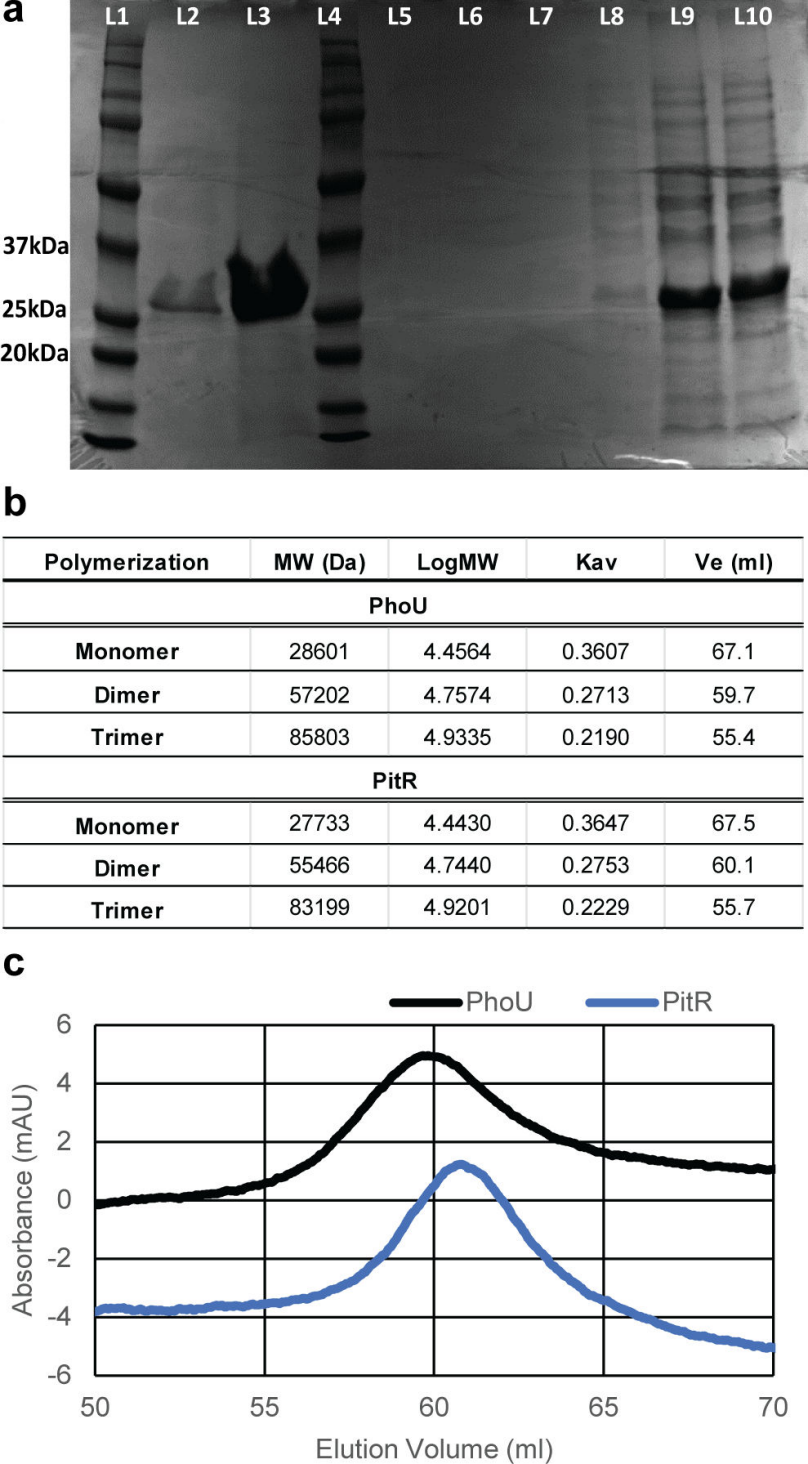
PhoU and PitR expression, purification, and size exclusion chromatography. (**a**) SDS-PAGE image of PhoU and PitR. L1 and L4 are protein markers; L2 and L3 are Ni-NTA-purified PhoU and PitR, respectively; and L9 and L10 are cell lysate of PhoU and PitR, respectively. (**b**) Using standard curve data, the expected elution volumes (Ve) of various PhoU protein combinations were calculated. (**c**) Purified PhoU and PitR proteins were run independently on the same column and eluted at the expected sizes for homodimers.

To further characterize the PhoU proteins from *S. aureus*, we cloned *phoU* and *pitR* genes in an expression vector with an added amino terminal histidine tag. Proteins were expressed and purified with a nickel-affinity column (Fig. 3a). Purified protein was then run on a size exclusion column to identify the size of purified protein complexes. Purified PhoU protein eluted at a volume of 59.8 mL, and PitR protein eluted at a volume of 60.8 mL (Fig. 3c). Based on calculated elution volumes, both PhoU- and PitR-purified proteins elute at a volume consistent with dimer formation (calculated to be 59.7 mL and 60.1 mL, respectively) (Fig. 3b and c).

## DISCUSSION

*S. aureus* PhoU homologs showed interaction with themselves, but do not appear to interact with PhoR or PstB. *E. coli* PhoU interacts with PhoR, PstB, and PhoU proteins when the genes are expressed in a BACTH (6). While most of the combinations of different proteins were negative for significant interaction, seeing some interactions with PhoU, PitR, and PhoR constructs implies that these proteins are successfully expressed and folded to some extent. However, it is possible that there were other factors that lead to no significant interaction in some of these combinations (like poor protein expression, folding, etc.). Limited interpretation should be based on these negative results.

We hypothesized that a PhoU homolog might interact with a different transporter complex than PstSCAB. However, we did not identify any interaction of PhoU or PitR with the other *S. aureus* phosphate transporters, PitA or NptA. Another possibility could be that NptA, as a transporter with a PhoU-like domain, may interact with PhoR independent of PhoU and/or PstB. However, our data did not show any significant NptA/PhoR interaction either.

Other studies have found that *S. aureus* can adapt to phosphate limitation through scavenging wall teichoic acids from other bacteria (30). Clearly, the mechanisms for adapting to changes in phosphate availability are not fully conserved across all bacterial species.

Concerning persister formation, the role of PhoU proteins may be regulatory, where a PhoU potentially interacts with other histidine kinases to alter gene expression. Alternatively, the effect of PhoU may be indirect, possibly through PhoU altering general cell fitness. Notably, phosphate ions were found to influence the binding of daptomycin to *Enterococcus faecalis* (31). This suggests that the potential action of a PhoU homolog altering the concentration of available phosphate ions may alter the effectiveness of daptomycin treatment in some bacteria.

Based on the BACTH results, it appears that each PhoU homolog interacts with itself. These results imply that PhoU homodimers may form, similar to other PhoU proteins (6, 23). The strongest protein/protein interaction was seen between PhoU and PhoU. The corresponding dimer model shows tight interaction between the two proteins with several predicted hydrogen bonds forming between the two PhoU proteins. Additionally, the PitR/PitR interaction was significant in the BACTH assay, and the predicted dimerization model shows potential interaction. However, the model does not predict any hydrogen bonding between the proteins, which may explain the relatively weaker protein/protein interaction observed in the BACTH results. Any PhoU/PitR interaction probed with the BACTH system was weak and not significant. The lack of PhoU/PitR interaction may be somewhat expected, given that the proteins do not have fully redundant functions (17). If the proteins were interchangeable, then one might expect heterodimerization as well as homodimerization. These results further confirm the importance of not just relying on sequence similarity when characterizing proteins.

While these dimer models correlate with the BACTH data, they are still only predictions. However, analyses of purified proteins using size exclusion columns confirmed the formation of dimers for both PhoU and PitR. These results are similar to the PhoU protein of *E. coli*, indicating common structural features across different species for PhoU proteins, despite their potential different functions in the different species (6). Perhaps the PhoU proteins in *S. aureus* have evolved from regulating phosphate response and transport to regulating other gene expression using similar mechanisms that involve dimer formation.

### Conclusion

These results reveal that neither of the *S. aureus* PhoU homologs appears to interact entirely like *E. coli*’s PhoU, but they do appear to interact with themselves. It is possible that *S. aureus* PhoU proteins may interact with other proteins in their roles in regulating persister formation and potentially affecting virulence factor expression. Additionally, dimerization models were identified, and the dimerization of each PhoU protein was confirmed with size exclusion chromatography. Further research to identify these regulatory mechanisms could reveal novel targets for future treatments. These results confirm the previous conclusions that these two proteins are distinct and the PhoU proteins form dimers. This study provides more evidence of variation in how each bacterial species may regulate gene expression, even when the species have a complement of very similar proteins. These insights further identify the importance of studying specific pathogenic bacteria when working to develop strategies to inhibit essential functions, as those functions may vary widely among different bacterial stains.

## MATERIALS AND METHODS

### Bacteria strains, plasmids, and growth conditions

Interactions between proteins were analyzed using the BACTH kit (EuroMedex) plasmids pKT25 and pUT18C in the *E. coli* BTH101 strain. Cultures were grown on Luria-Bertani (LB) agar plates, and when necessary, media was supplemented with antibiotics: ampicillin (100 µg/mL), kanamycin (50 µg/mL), and isopropyl β- d-1-thiogalactopyranoside (IPTG) (0.5 mM). *E. coli* was cultured at 37°C overnight with shaking. For the BACTH constructs, *S. aureus* Mu50 genomic DNA was used for the PCR amplification of DNA fragments with primers that incorporated the restriction sites of XbaI and KpnI for *pitA*, *pstB*, *phoR*, *phoU*, and *pitR* (but with PstI and KpnI for *nptA*, due to an XbaI site found inside the *nptA* gene). Primers used for PCR are listed in Table 1. PCR reactions consisted of 2.5 µL of 10 µM forward primer, 2.5 µL of 10 µM reverse primer, 5 µL of *S. aureus* Mu50 DNA, 2.5 µL nuclease-free water, and 12.5 µL of Bullseye Taq Pro 2 × Master Mix (MidSci). PCR fragments were cleaned using Wizard SV Gel and PCR Clean-Up System (Promega) and checked for amplification with an agarose gel. Plasmids and PCR products were digested for 2 hours at 37°C to create sticky ends using 1 µL of each restriction enzyme, 5 µL of 10 × CutSmart buffer (New England BioLabs), 15 µL DNA, and 28 µL of water. Antarctic Phosphatase (New England BioLabs), 2.5 µL, and Antarctic Phosphatase buffer, 5 µL, were added to digested plasmids, incubated for 30 minutes at 37°C, and inactivated by heating to 80°C for 2 minutes. Ligation reactions used 1 µL T4 DNA ligase (New England BioLabs), 2 µL T4 DNA ligase buffer, 1 µL digested plasmid, and 2.5 µL digested PCR fragment in 13.5 µL of water at 16°C for 2 hours or overnight.

**TABLE 1:** List of primers^*a*^

Primer name	Sequence in 5′ to 3′ direction
NptApKTFor	GT**CTGCAG**TAATGGAAATGTCGGTTACAGAAGT
NptApUTFor	CA**CTGCAG**AATGGAAATGTCGGTTACAGAAGT
NptARev	GGTA**GGTACC**TTATTTTCAGTTGTTGCAATTTCTTC
PhoRFor	AC**TCTAGA**GATGATGAAGTTTCACCACCG
PhoRRev	GGTA**GGTACC**TTTTCTTTATAATCTTTTAGAATAACTTTGAA
PhoUFor	AC**TCTAGA**GATGGCAATAATTAGACAACGATATCAG
PhoURev	GGTA**GGTACC**TTTTGTTCGTAATGTGTACCTGT
PitRFor	AC**TCTAGA**GATGTTTAGTAAGAAAAAAGATAAGTTTATGGTT
PitRRev	GGTA**GGTACC**TTGCTATTTTTCATAATAATAGTTTCAAAATT
PitAFor	AC**TCTAGA**GATGTCATATATAATCATCGTCACTATAGCT
PitARev	GGTA**GGTACC**TTGAAAAATAAGTTAAGTATATAGAATAG
PstBFor	AC**TCTAGA**GATGGCGCAAACACTTGCACAA
PstBRev	GGTA**GGTACC**TTACCAAACCTTCCTGAAAT
pKT25For	TCCAACTTCCGCGACTC
M13-fwd	TGTAAAACGACGGCCAGT
pUT18CFor	CGAAGTTCTCGCCGGATG
pUT18CRev	GGCTTAACTATGCGGCATC
PhoUHCF	TAAGGCCTCTGTCGAATGGCAATAATTAGACAGCGATA
PhoUHR	CAGAATTCGCAAGCTCTTGTTCGTAATGTGTACCTGTTA
PitRHCF	TAAGGCCTCTGTCGAATGTTTAGTAAGAAAAAAGATAAGT
PitRHR	CAGAATTCGCAAGCTCGCTATTTTTCATAATAATAGTTTCA

^*a*^ DNA primers used for PCR amplification of inserts into the BACTH plasmids; bolded letters represent endonuclease sites added for ease of cloning. DNA primers used for sequencing confirmation (pKT25For, M13-fwd, pUT18CFor, and pUT18CRev), and DNA primers used for cloning into the pET6xHN vector (PhoUHCF, PhoUHR, PitRHCF, and PitRHR).

*E. coli* BTH101-competent cells were prepared by diluting an overnight culture 1:100 into 25 mL of fresh LB media in a 250-mL flask, grown at 37°C with shaking at 200 rpm until it reached an OD_600_ of about 0.4, and collected by centrifuging for 10 minutes at 5000 rpm at 4°C. The supernatant was removed, the pellet was resuspended in 5 mL of cold 0.1 M CaCl_2_, cells were incubated on ice for 15–30 min, then cells were centrifuged again, the supernatant was removed, and the pellet was resuspended in 0.8 mL of 0.1 M CaCl2 with 15% glycerol. Competent cells were stored in 0.1-mL aliquots at −80°C.

For transformation of plasmids, 0.1 mL of competent cells were thawed on ice for 30 minutes, and 2.5 µL of plasmid DNA was added, incubated on ice for 30 minutes, heat-shocked in a 42°C water bath for 75 seconds, and returned to ice for 2 minutes. 0.9 mL of LB media was added to each tube and then incubated for 1 hour at 37°C with shaking at 200 rpm. Transformants were plated onto LB agar containing the appropriate antibiotics, incubated overnight at 37°C, and isolated colonies were then streaked on LB agar with antibiotics.

Plasmids were purified using a ZR plasmid miniprep kit (Zymo Research), and successful insertion of genes into each plasmid was checked by running digested plasmid samples on a 2% agarose gel and confirmed by DNA sequencing (MCLabs) using sequencing primers listed in Table 1 (data not shown).

For protein expression, the pET6xHN vector (Takara Bio) with PhoU and PitR inserts were incorporated separately into the prelinearized expression vector. *phoU* and *pitR* genes were amplified from the *S. aureus* Mu50 genomic DNA. For incorporation of PCR inserts into the vector, we used 2 µL 5X In-Fusion HD Enzyme Premix, 1 µL linearized vector (In-Fusion ready pET6xHN), 2 µL PCR insert, and 5 µL water. The reaction mixture was incubated for 15 minutes at 50°C and then placed on ice. Samples were then transformed into *E. coli* BL21 (DE3) cells.

### β-galactosidase assay

Protein/protein interactions were measured with a modified β-galactosidase activity assay as described in the Euromedex BACTH system kit. *E. coli* cells were grown in 5 mL of LB with 0.5 mM IPTG, 100 µg/mL ampicillin, and 50 µg/mL kanamycin at 30°C and shaken at 250 rpm overnight. Cultures were vortexed for 5 seconds and diluted 1 to 5 with LB medium, and the OD_600_ was determined with a Thermo Scientific Multiskan FC 96-well plate reader. Cells were permeabilized by adding two drops of chloroform and one drop of a 0.1% SDS solution per 2.5 mL of the diluted cell suspensions. The tubes were vortexed for 10 seconds and then vigorously agitated in a shaker at 37°C for 30 minutes. 0.2 mL of the permeabilized cells were added to 0.8 mL of PM2 buffer (70 mM Na_2_HPO_4_ · 12 H_2_O, 30 mM NaH_2_PO_4_ · H_2_O, 1 mM MgSO_4_, 0.2 mM MnSO_4_ solution at pH 7.0) with 100 mM of β-mercaptoethanol added just before use, in 5-mL tubes. The tubes were placed in a water bath at 28°C for 5 minutes. The reactions were started by adding 0.25 mL to the o-nitrophenyl-β-galactoside (ONPG) substrate solution (0.4% ONPG in PM2 buffer without β-mercaptoethanol) pre-equilibrated at 30°C. After a sufficient yellow color developed, 0.5 mL of the 1 M Na_2_CO_3_ was added. The time from the addition of ONPG to reaction stoppage with Na_2_CO_3_ was recorded, and the OD_420_ was recorded. Activity units are equal to 200 × (OD_420_ − OD_420_ in control tube) / minutes of incubation) × dilution factor, 1:5. Results are reported as units/mg of dry-weight bacteria, which is equal to units × 1,000 / 300 µg × OD_600_. OD_600_ was blanked with LB broth. OD_420_ was blanked with 1 mL PM2 buffer, 0.25 mL ONPG, and 0.5 mL Na_2_CO_3_.

Each BACTH sample was tested multiple times, and representative results were reported. The PhoU, PitR, NptA, PstB, and PhoR expressing pKT25 plasmids were each co-transformed with the complimentary pUT18C plasmids. All the combinations for a single pKT25 plasmid were tested at the same time in a single experiment, including the controls. Each combination was tested with four biological replicates (independent bacterial colonies). Each biological sample had three technical replicates to get a sample mean. Data are displayed as the mean ± SD and analyzed with a one-way ANOVA and a Dunnett’s multiple comparisons test for each sample compared to the negative control sample from that experiment (using Prism 10 [GraphPad Prism Version 10.2.3 (403]).

### Protein expression

*E. coli* BL21 cells with either pETphoU1_6xHN or pETphoU2_6xHN plasmids were grown at 37°C overnight with shaking in 5 mL LB broth with 100 µg/mL ampicillin. The primary culture was diluted 1:100 into 35 mL of fresh LB media in a 250-mL conical flask with 100 µg/mL ampicillin. The fresh culture was incubated for 4 hours with shaking and then supplemented with 1 mM IPTG and incubated for a further 2 hours.

### Protein purification

The bacterial culture was centrifuged at 5,000×*g* for 20 minutes at 4°C. The pellet was resuspended in 8 mL xTractor Buffer (Takara Bio) and shaken at 200 rpm for 15 minutes at 4°C. The mixture was centrifuged at 13.3×*g* for 20 minutes at 4°C. 1 mL His60 Ni gravity columns (Takara Bio) were pre-equilibrated with 5 mL lysis buffer (10 mM NaH_2_PO_4_/Na_2_HPO_4_, 0.5 M NaCl, 20 mM imidazole, pH 7.4). After loading the sample into the column, it was kept on ice for 5 minutes to facilitate the binding of histidine-tagged protein to nickel. The column was washed with 5 mL of the same lysis buffer. To elute protein, 3 mL of elution buffer (20 mM NaH_2_PO_4_, Na_2_HPO_4_, 0.5 M NaCl, 500 mM imidazole, pH 7.4) was used.

### SDS-PAGE analysis

The presence of desired protein was assessed using SDS-PAGE gel with precast Mini-PROTEAN TGX 12% gels (Bio-Rad). The gel was suspended in a 1 × running buffer (Tris-OH, Glycine, SDS, pH 8.3). Samples were added in equal volumes of 2 × Laemmli buffer with 5% 2-mercaptoethanol and heated in a dry bath at 95°C for 5 minutes before loading into a gel. Five microliters of Precision Plus Protein Dual Color Standards (Bio-Rad) was used. Twenty microliters of each sample was added to the SDS-PAGE and ran for approximately 45 minutes at 160V. Gel was stained in a solution of 50% methanol, 10% acetic acid, and 40% water with 0.25% Coomassie Blue R-250 overnight. The gel was destained overnight in a solution of 5% methanol, 7.5% acetic acid, and 87.5% H_2_O. The gels were imaged using a ProteinSimple FluorChem E System.

### Size exclusion chromatography

A HiPrep 16/60 Sephacryl S-200 HR size-exclusion column mounted on AKTA Purifier chromatography system (GE Healthcare) was used. Prior to sample loading, the column was equilibrated with a buffer consisting of 50 mM NaPO_4_ and 150 mM NaCl pH 7.0. The sample was allowed to pass through the column at a rate of 0.5 mL/min. Protein standards (Sigma-Aldrich) were run under the same conditions to create a calibration curve and used to measure the size of PhoU1 and PhoU2. Using the equation for the line that fit the protein standard data (Kav = −0.2971(logMW) + 1.6847) with 37.3 mL as void volume (based on the volume to elute a sample of blue dextran), we used the equation Ve = (Kav × 82.7) + 37.3 to calculate expected elution volumes of various PhoU protein combinations. PhoU and PitR were separately loaded immediately after purification.

### Structure prediction models

Protein sequences for the Mu50 strain of *S. aureus* and the MG1655 strain of *E. coli* were accessed using the Kyoto Encyclopedia of Genes and Genomes (32). The PhoU sequence from *Pseudomonas aeruginosa* was accessed from the protein data bank website (https://www.rcsb.org/structure/4Q25). PhoU protein sequences from *E. coli*, *S. aureus*, and *P. aeruginosa* were aligned and compared using Clustal Omega (33). The three-dimensional structures of PhoU and PitR subunits were predicted using Phyre2 (28, 34). PhoU/PhoU and PitR/PitR dimer models were predicted using protein/protein docking modeling with ClusPro (29, 35–37). Predicted structures were displayed using the software program ChimeraX (UCSF).

## Data Availability

The data that support the findings of this study are openly available in FigShare (http://doi.org/10.6084/m9.figshare.24128505). The protein structure models are available in ModelArchive (modelarchive.org) with the accession codes: ma-6hipq, ma-x4rb5, ma-jqp3h, and ma-cqprc.
